# Pulmonary arterial dysfunction in insulin resistant obese Zucker rats

**DOI:** 10.1186/1465-9921-12-51

**Published:** 2011-04-22

**Authors:** Javier Moral-Sanz, Carmen Menendez, Laura Moreno, Enrique Moreno, Angel Cogolludo, Francisco Perez-Vizcaino

**Affiliations:** 1 Departamento de Farmacologia, Facultad de Medicina, Universidad Complutense de Madrid, 28040 Madrid. Spain and Ciber Enfermedades Respiratorias, CIBERES

## Abstract

**Background:**

Insulin resistance and obesity are strongly associated with systemic cardiovascular diseases. Recent reports have also suggested a link between insulin resistance with pulmonary arterial hypertension. The aim of this study was to analyze pulmonary vascular function in the insulin resistant obese Zucker rat.

**Methods:**

Large and small pulmonary arteries from obese Zucker rat and their lean counterparts were mounted for isometric tension recording. mRNA and protein expression was measured by RT-PCR or Western blot, respectively. K_V _currents were recorded in isolated pulmonary artery smooth muscle cells using the patch clamp technique.

**Results:**

Right ventricular wall thickness was similar in obese and lean Zucker rats. Lung BMPR2, K_V_1.5 and 5-HT_2A _receptor mRNA and protein expression and K_V _current density were also similar in the two rat strains. In conductance and resistance pulmonary arteries, the similar relaxant responses to acetylcholine and nitroprusside and unchanged lung eNOS expression revealed a preserved endothelial function. However, in resistance (but not in conductance) pulmonary arteries from obese rats a reduced response to several vasoconstrictor agents (hypoxia, phenylephrine and 5-HT) was observed. The hyporesponsiveness to vasoconstrictors was reversed by L-NAME and prevented by the iNOS inhibitor 1400W.

**Conclusions:**

In contrast to rat models of type 1 diabetes or other mice models of insulin resistance, the obese Zucker rats did not show any of the characteristic features of pulmonary hypertension but rather a reduced vasoconstrictor response which could be prevented by inhibition of iNOS.

## Background

Pulmonary arterial hypertension (PAH) is a progressive disease of poor prognosis characterized by vasoconstriction of pulmonary arteries (PA) and proliferation of pulmonary vascular endothelial and smooth muscle cells leading to increase vascular resistance and right heart failure with right ventricular hypertrophy as a hallmark [1,2]. These pathological events are influenced by genetic predisposition as well as environmental stimuli [1,3]. Bone Morphogenetic Protein Receptor 2 (BMPR2) gene mutations have been described in some PAH patients [4] and diminished expression of its encoded protein has also been shown in both human and animal models of PAH [5-8]. Additionally, endothelial dysfunction and increased 5-HT contractile response have been reported in PAH [9-11]. Several studies have reported the involvement of K_V _channels in controlling membrane potential of pulmonary artery smooth muscle cells (PASMC) and PA tone [12]. Moreover, it was reported the role of K_V_1.5 in the development of PAH as a result of mutation or downregulation of the channel [13,14].

Obesity and insulin resistance have a worldwide increasing prevalence. Despite the fact that insulin resistance is strongly associated with systemic cardiovascular diseases [15,16] the relationship with pulmonary vascular disease has been almost disregarded [17]. Recent reports have suggested that insulin resistance might also be associated with pulmonary hypertension in humans [18-20] and in the ApoE deficient mice [21]. In rats with type 1 diabetes, we have recently found pulmonary endothelial dysfunction associated to increased superoxide production and upregulation of the NADPH oxidase subunit p47^phox ^[8]. The Obese Zucker rat is a well establish model of obesity and insulin resistance associated to systemic vascular dysfunction [22-24]. Nonetheless, the pulmonary vasculature remains uncharacterized in this model. Therefore, the present study was designed to analyze the pulmonary markers of PAH including the pulmonary expression of key proteins of the disease, K_V _currents, vascular reactivity of PA, and right ventricular hypertrophy in obese Zucker rats compared to their lean Zucker littermates.

## Methods

### Ethics statement

The present investigation conforms to the *Guide for the Care and Use of Laboratory Animals *(National Institutes of Health Publication No. 85-23, revised 1996), and the procedures were approved by our institutional review board (Comité de Experimentación Animal, Universidad Complutense, 070208).

### Animals, tissues and reagents

On the day of the experiment, male obese Zucker rats (fa/fa) and their littermates, lean Zucker rats (fa/-) (17-18 weeks old) were weighed and sacrificed by cervical dislocation and exsanguination. Pulmonary arteries (PA) were dissected to obtain conductance and resistance intrapulmonary arteries. Smooth muscle cells were then enzymatically isolated from resistance intrapulmonary arteries [25]. Blood glucose was measured using a clinical glucometer (OneTouch Ultra) and insulin using an enzyme immunoassay. Hearts were excised, fixed with formol embedded in paraffin and cut into 1 mm cross sections, visualized in a microscope, photographed and analyzed using imageJ (Ver 1.41, NIH, USA). All drugs were from Sigma (Tres Cantos, Spain).

### Vascular reactivity

Resistance (diameter ~0.3-0.5 mm and length ~2 mm) and conductance (diameter ~1-1.2 mm and length ~3 mm) PA rings were mounted in Krebs solution at 37°C gassed with a 95% O_2_-5% CO_2 _mixture in a wire myograph or in organ chambers respectively. After stretching to give an appropriate resting tension (equivalent to 30 mm Hg as previously described [25] for resistance or 0.7 g for conductance arteries) each vessel was exposed to different vasoconstrictor agents to test the vascular response. The contractile responses were performed by cumulative addition and expressed as a percentage of the response to 80 mM KCl. The endothelial function was estimated by the analysis of the relaxant response to cumulative addition of acetylcholine (ACh, 10^-9^-10^-4^M) after precontraction with 10^-7^M phenylephrine in conductance arteries or with a concentration of phenylephrine titrated to induce a contraction 75% of the response to KCl. Some experiments were carried out in the presence of the NOS inhibitor L-NAME. Hypoxia was induced by bubbling the Krebs solution with 95%N_2_-5% CO_2 _to achieve an oxygen concentration of 3-4% (24 ± 1 Torr) in the chamber as described [26].

### Electrophysiological studies

Membrane currents were recorded using the whole-cell configuration of the patch clamp technique with an Axopatch 200B and a analog to digital converter Digidata 1322A (Axon Instruments, Burlingame, CA, U.S.A). pClamp version 9 software was used for data acquisition and analysis. Cells were superfused with an external Ca^2+^-free Hepes solution (2 ml/min) and a Ca^2+^-free pipette (internal) solution containing (mmol/L): KCl 110, MgCl_2 _1.2, Na_2_ATP 5, HEPES 10, EGTA 10, pH adjusted to 7.3 with KOH. Patch pipettes (2-4 MΩ) were constructed from borosilicate glass capillaries (GD-1, Narishige Scientific Instruments, Tokyo, Japan) using a programmable horizontal puller. Currents were evoked following the application of 200 ms depolarizing pulses from -60 mV to test potentials from -60 mV to +60 mV in 10 mV increments [27]. Hypoxia was induced by bubbling the solution with N_2 _as described [26].

### Protein expression

Whole lungs were homogenated under reducing conditions in the presence of DTT, proteases and phosphatases inhibitors. Protein content was determined by Bio-Rad DC Protein Assay Kit (Bio-Rad, Hercules, CA, USA) and equal amounts of proteins were loaded and subjected to electrophoresis on a SDS-PAGE (7.5-10%) followed by a transference to a PVDF membrane (Bio-Rad). Protein expression was quantified using primary antibodies anti-K_V_1.5 (Alomone, Israel, 1:200 dilution), anti-5HT_2A _(BD Biosciencies, 1:250 dilution), anti- Bone Morphogenetic Protein Receptor 2 (BMPR2) (BD Biosciencies, 1:250 dilution), anti-eNOS (BD Biosciencies, 1:2500 dilution), anti-iNOS (Santa Cruz, CA, USA, 1:500 dilution), anti-β-actin (Sigma-Aldrich, Spain, 1:5000 dilution) and horseradish peroxidase conjugated secondary goat anti-mouse and anti-rabbit antibodies (Santa Cruz Biotech, CA, USA, 1:10000 dilution). Proteins were detected using ECL-Plus Western blotting reagents (Amersham, GE Healthcare, CT, USA) and analyzed using Quantity One (BioRad).

### Real time RT-PCR

Total RNA was isolated and purified from resistance PA homogenates using RNeasy Mini kit (Qiagen, Hilden, Germany) and converted into cDNA using iScript cDNA synthesis kit (BioRad, Hemel Hempstead, UK). Real-time PCR was performed using a Taqman system (Roche Diagnostics, Mannheim, Germany) in the Genomic Unit of Universidad Complutense de Madrid. Specific primers were designed for rat K_V_1.5 (sense 5'-GGAAGAACAAGGCAACCAGA-3', antisense 5'-AGCTGACCTTCCGTTGACC-3'), iNOS (sense 5'-TTGGAGTTCACCCAGTTGTG-3', antisense 5'-ACATCGAAGCGGCCATAG-3'), eNOS (sense 5'-GGTATTTGATGCTCGGGACT-3', antisense 5'-TGTGGTTACAGATGTAGGTGAACA-3'), BMPR2 (sense 5'-CGGGCAGGATAAATCAGGA-3', antisense 5'-CAGGAAAGTAAATTCGGGTGA-3') and β-actin (sense 5'-GCCCTAGACTTCGAGCAAGA-3', antisense 5'-TCAGGCAGCTCATAGCTCTTC-3'). Data were normalized by the expression of β-actin.

### Statistical analysis

Results are expressed as mean ± s.e.m. Data for Western blots and RT-PCR were normalized by the expression of β-actin and expressed as a percentage of the values obtained in the lean rats. Individual cumulative concentration-response curves were fitted to a logistic equation. The negative logarithm of the molar concentration that causes 50% of the maximum response (pD_2_) and the maximum response (E_max_) were calculated for each ring. Statistical analysis was performed by comparing the lean and obese Zucker groups with an unpaired Student's *t*-test. Differences were considered statistically significant when *P *< 0.05.

## Results

Obese Zucker rats showed a final body weight ~30% higher than their lean littermates (476 ± 29 vs 364 ± 22 g, respectively, P < 0.01, n = 20 for both groups). Non fasting blood glucose was not significantly different (128 ± 13 vs 106 ± 5 mg/dL, respectively, n = 13 and 12) but insulin was strongly elevated (3.5 ± 0.2 vs 1.4 ± 0.2 ng/ml, respectively, n = 7 for both groups).

### Heart wall thickness and BMPR2 expression

**Figure 1:**
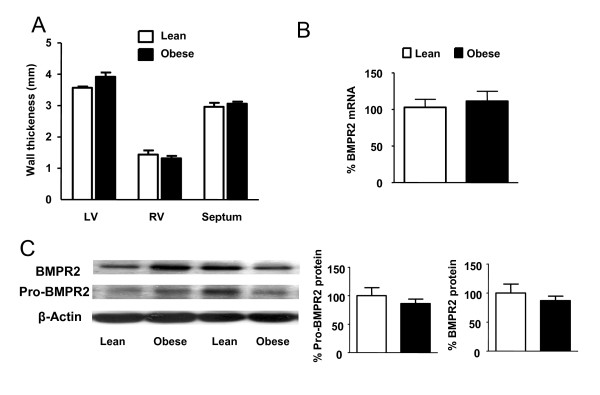
**Heart wall thickness and BMPR2 expression**. (A) Left ventricular (LV), right ventricular (RV) and septal wall thickness from lean (n = 8) and obese (n = 7) Zucker rats. (B) BMPR2 mRNA expression in resistance PA of lean and obese (n = 5) analyzed by RT-PCR and normalized by β-actin expression. (C) BMPR2 precursor (~115 KDa) and mature (~75 KDa) protein expression from obese and lean Zucker lungs (n = 8) analyzed by Western blot and normalized by β-actin expression. Results indicate mean ± s.e.m.

No significant changes were found in the wall thickness of the right ventricle (RV), the left ventricle (LV) or the septum (S) from obese as compared with lean rats (Figure 1A). The RT-PCR analysis revealed no changes in mRNA transcription levels of BMPR2 gene in resistance PA (Figure 1B) and Western blots showed no significant changes in the whole lung protein expression of BMPR2 or in its heavier precursor (pro-BMPR2) (Figure 1C).

### K_V _currents and K_V_1.5 lung expression

**Figure 2:**
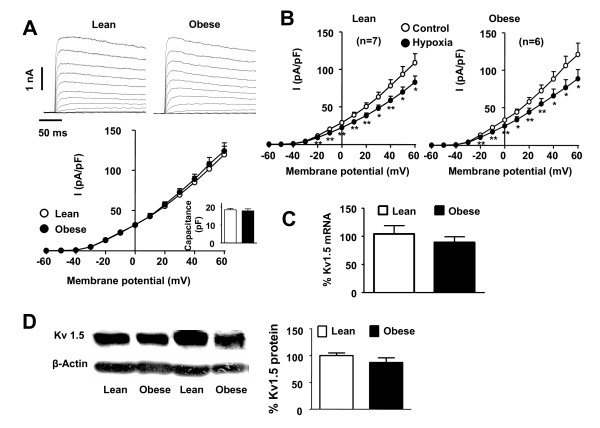
**K_V _currents and K_V_1.5 expression**. (A) K_V _current traces recorded in enzymatically isolated PASMC from lean and obese Zucker rats with depolarizing pulses from -60 mV to +60 mV in 10 mV increments. The current-voltage relationship measured at the end of depolarizing pulse is shown at the bottom (n = 9) and the membrane capacitance in the inset. (B) Effects of hypoxia on Kv currents in both strains (n = 7). (C) K_V_1.5 mRNA expression in resistance PA from lean and obese Zucker rats analyzed by RT-PCR and normalized by β-actin expression (n = 5). (D) K_V_1.5 protein expression in whole lung homogenates analyzed by Western blot and normalized by β-actin expression (n = 6). Results indicate mean ± s.e.m.

Similar cell capacitance (17.8 ± 1.1 and 18.4 ± 0.7 pF in obese and lean rats, respectively), as a measure of the cell size, and similar K_V _current density (Figure 2A) were found in lean and obese PASMC. Moreover, hypoxia induced a similar inhibition of K_V _currents in both strains (Figure 2B). In accordance with patch-clamp data, no changes in K_V_1.5 mRNA transcription in resistance PA (Figure 2C) or whole lung protein expression (Figure 2D) were found in obese as compared to lean rats.

### Endothelial function

**Figure 3:**
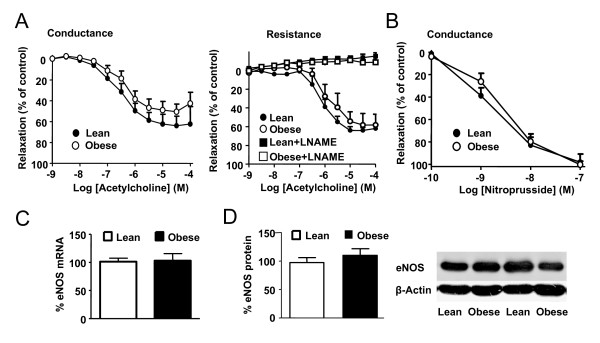
**Endothelial function and eNOS protein expression**. (A) Concentration-response curve to acetylcholine in endothelium intact conductance PA rings precontracted with phenylephrine 10^-7^M (left) and resistance PA rings precontracted with phenyleprine to reach a 75% of KCl contraction with or without L-NAME 10^-4 ^M (right) from lean and obese Zucker rats (n = 4-6). (B) Concentration-response curve to sodium nitroprusside in conductance PA rings contracted by 5-HT (10^-4^M) in the presence of L-NAME (10^-4^M, n = 5). (C) eNOS mRNA levels in resistance PA analyzed by RT-PCR and normalized by β-actin expression (n = 5) and (D) eNOS protein expression from whole lung homogenated analyzed by Western blot and normalized by β-actin expression (n = 8). Results indicate mean ± s.e.m.

The endothelial function was tested in endothelium intact PA preconstricted with phenylephrine (10^-7^M in conductance arteries or a concentration titrated to induce a contraction 75% of the response to KCl in resistance PA). Increasing concentrations of ACh induced a similar relaxant response in obese and lean rats in conductance arteries (Figure 3A). Resistance arteries from obese rats required higher concentrations of phenylephrine to achieve a tone similar to the lean ones (5 ± 2 · 10^-6^M vs 7 ± 2 · 10^-7^M, respectively). The analysis of the concentration-response curves to ACh shows that there were not significant changes in the E_max _values between groups in conductance (E_max _53 ± 7 vs 67 ± 9%, respectively) or resistance vessels (E_max _59 ± 8 vs 66 ± 4%, respectively). Similarly, the concentration of ACh required for half-maximal relaxation in conductance (pD_2 _values 6.4 ± 0.1 vs 6.2 ± 0.2, respectively) or in resistance vessels (pD_2 _values 6.1 ± 0.2 vs 5.8 ± 0.2, respectively) was similar in both groups. In the presence of the NOS inhibitor L-NAME, similar concentrations of phenylephrine were required to induce ~75% of KCl contraction in arteries from the obese and lean rats (3 ± 2·10^-8^M and 2 ± 0.6·10^-8^M, respectively) but these concentrations were significantly lower than those required in the absence of L-NAME. Moreover, in the presence of this inhibitor, the relaxation to acetylcholine was completely abolished in both strains (Figure 3D). In addition, no changes were found in the response to the endothelium-independent vasodilator sodium nitroprusside in conductance PA (Figure 3B). Expression of eNOS mRNA in resistance PA (Figure 3C) or eNOS protein in whole lung (Figure 3D) was also similar in both strains.

### Contractile responses in conductance PA

**Figure 4:**
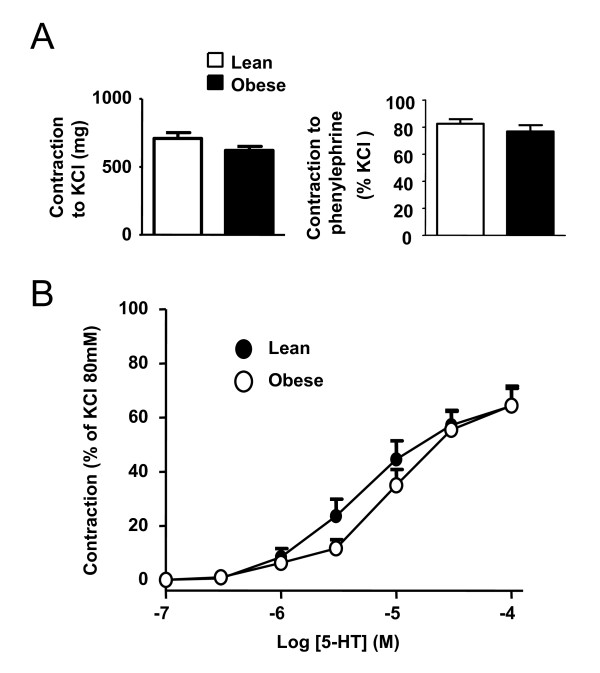
**Vasoconstrictor responses in conductance PA**. (A) Contractile responses to KCl (80 mM, n = 5, left) and phenylephrine (10^-7 ^M, n = 5, right) in conductance PA from lean and obese Zucker rats. (B) Serotonin concentration-response curve in conductance PA from lean and obese Zucker rats. Results indicate mean ± s.e.m.

Conductance pulmonary arteries were mounted in organ chambers to test the contractile response to 80 mM KCl, phenylephrine and 5-HT. No changes were found in the responses to the vasoconstrictor agents KCl (80 mM) or phenylephrine (10^-7^M) when both groups of rats were compared (Figure 4A). A similar concentration-response curve to 5-HT was also obtained in obese and lean rats (Figure 4B, E_max _and pD_2 _values are shown in Table 1).

**Table 1:** Parameters of the concentration-response curve to vasoconstrictor agonists in isolated conductance and resistance PA from lean and obese Zucker rats [means ± s.e.m. (n)].

	**E**_**max**_**(% of KCl)**		**pD**_**2**_	
	
	Lean	Obese	Lean	Obese
**Conductance PA**				

**Phenylephrine**	82.6 ± 3.2 (5)	83.7 ± 4.1(5)	8.20 ± 0.03	8.10 ± 0.09

**5-HT**	64.3 ± 6.5 (5)	64.4 ± 7.2(5)	5.28 ± 0.13	5.02 ± 0.11

**α-methyl-5-HT**	41.0 ± 9.5 (6)	30.8 ± 6.8(6)	5.32 ± 0.13	5.46 ± 0.12

**Resistance PA**				

**5-HT**	69.2 ± 7.8 (6)	33.5 ± 9.1 * (6)	5.28 ± 0.10	4.86 ± 0.06 **

**5-HT (1400W)**	49 ± 7 (6)	58 ± 9 (6)	5.10 ± 0.13	5.24 ± 0.15

**α-methyl-5-HT**	33.7 ± 8.8 (4)	9.5 ± 3.6 * (4)	5.80 ± 0.11	5.58 ± 0.10

### Contractile responses in resistance PA

**Figure 5:**
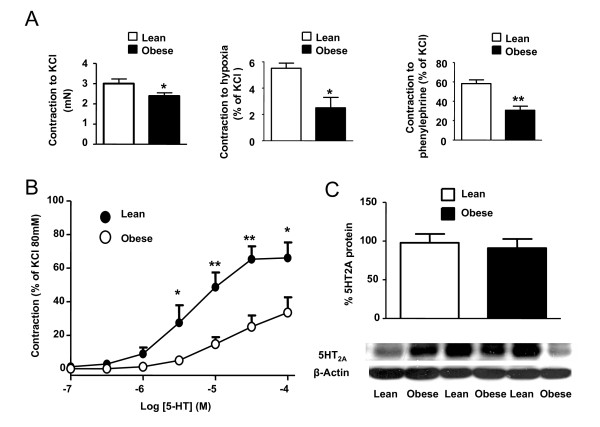
**Vasoconstrictor responses in resistance PA**. (A) Contractile responses of resistance PA induced by KCl (80 mM, n = 8, left), hypoxia (n = 3, middle) and phenyleprine (10^-7 ^M, n = 3-4, right) in resistance PA from lean and obese Zucker rats. (B) Concentration-response curve to 5-HT (n = 6). (C) Whole lung protein expression of 5-HT_2A _receptor (n = 8). Results indicate mean ± s.e.m. *, ** denote P < 0.05 and P < 0.01 respectively, obese vs lean.

The contractile response to 80 mM KCl in resistance PA showed a significant reduction in obese compared to lean rats. Obese rats also evidenced a significant hyporesponsiveness to hypoxia, phenylephrine and 5-HT (Figure 5 and Table 1). We further investigated the response to the 5-HT_2 _agonist α-methyl-5-HT. This agonist also showed reduced vasoconstriction responses in PA rings from obese rats (Table 1). Western blot analysis of whole lung homogenates revealed no changes in the expression of 5-HT_2A _receptors.

### Role of inducible NO synthase

**Figure 6:**
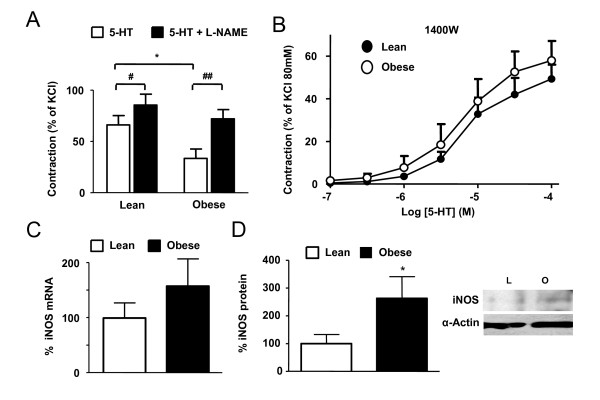
**Role of iNOS**. (A) Constrictor effect of 5-HT (10^-4^M) and the additional contractile effect of L-NAME (10^-4^M) on top of the response to 5-HT in resistance PA from lean (n = 7) and obese (n = 6) Zucker rats. (B) Concentration-response curves to 5-HT in the presence of 1400W (10^-5^M, n = 6) in resistance PA. (C) iNOS mRNA transcript levels in resistance PA (n = 6), (D) iNOS protein in resistance PA (n = 5 and 6, respectively). Results indicate mean ± s.e.m. * denotes P < 0.05 (obese vs lean, unpaired t test) and # and ## denote P < 0.05 and P < 0.01, respectively (pre vs post L-NAME paired t test).

To test the role of NO in the vascular hyporesponsiveness observed in resistance PA, the NO synthase inhibitor L-NAME was added on top of the maximal response to 5-HT. L-NAME induced a further contraction in both arteries but it was significantly higher in the obese rats. Therefore, no differences were found in the final tone induced by 5-HT plus L-NAME when both groups were compared, i.e. L-NAME restored the vascular hyporesponsiveness to 5-HT (Figure 6A). Interestingly, the incubation of the PA ring in the presence of the iNOS selective inhibitor 1400W prevented the reduced response to 5-HT observed in the PA from obese rats and thus the responses were similar in obese and lean rats (Figure 6B). These results suggest that iNOS might be a source of the NO responsible of the vascular hyporesponsiveness in the obese rats. The levels of iNOS mRNA expression were highly variable in the resistance PA from both groups and even when a trend to increased transcription of iNOS mRNA was observed, the difference did not achieve statistical significance (Figure 6C). However, we found a significant increase in iNOS protein expression in resistance pulmonary arteries from obese rats (Figure 6D).

## Discussion

Epidemiological studies show that insulin resistance appears to be more common in pulmonary hypertension than in the general population [18]. Similarly, patients with type II diabetes mellitus have significantly higher prevalence of pulmonary embolism and pulmonary hypertension independent of coronary diseases, hypertension, congestive hearth failure or smoking [19]. Recent data of our group demonstrated a marked endothelial dysfunction in PA characterized by an increase of reactive oxygen species and by an increased expression of p47^phox ^[8] as well as a decreased BMPR2 lung expression together with exaggerated response of PA to 5-HT (authors unpublished observations) in rats treated with streptozotocin as an insulin-dependent diabetes model. Additionally, experimental data demonstrated that ApoE^-/- ^mice on a high fat diet develop PAH as judged by an elevated right ventricular systolic pressure and augmented RV/(LV+S) relation when compared to controls [21]. The aim of the present study was to further investigate the relationship between insulin resistance and pulmonary hypertension. For this purpose we have used a well established genetic model of obesity and insulin resistance, the obese Zucker rat, characterized by a missense mutation in the leptin receptor [28] and associated with several cardiovascular complications [22,29].

Sustained elevated pulmonary pressure results in compensatory right ventricular hypertrophy and, therefore, the weight or the wall thickness of the right ventricle can be used as an indirect index of pulmonary artery pressure. Increased right ventricular weight compared to the left ventricle plus the septum weight has been described in streptozotocin-induced type 1 diabetes [30] and in insulin resistant ApoE knockout mice [21]. However, we did not find changes in the left or right ventricular wall thickness in obese Zucker rats as compared to lean ones. Fredersdorf et al. also reported similar heart weight in these strains [22]. Additionally, mutations in the BMPR2 or the diminished expression of BMPR2 has been described in lungs from PAH patients [4] and from rats with monocrotaline- or hypoxia-induced PAH [5-7]. Recently we also found a downregulation in the lung expression of BMPR2 in streptozotocin-treated rats (authors unpublished observations); nonetheless, our RT-PCR analysis revealed no changes in the BMPR2-mRNA levels of obese as compared to lean rats. This was further confirmed by Western blot analysis where the expression of neither BMPR2 nor its heavier precursor (pro-BMPR2) were significantly modified.

PAH has been associated with a decrease in PASMC K_V _currents and with reduced expression of K_V _channels, mainly K_V_1.5, K_V_3.1 and K_V_2.1 [14]. K_V_1.5 mRNA and protein expression, K_V _current density as well as the inhibitory effects of hypoxia in freshly isolated PASMC were unchanged in obese as compared to lean rats. Additionally, PASMC from obese rats showed no signs of hypertrophy as indicated by the capacitance data.

Endothelial dysfunction is characterized by a diminished vasodilator response to acetylcholine due to a reduced NO release or increase NO metabolism. Insulin resistant states and diabetes are associated to reduced endothelium-dependent relaxation and linked to cardiovascular events [31-33]. Moreover, endothelial dysfunction is a key factor in the development of retinopathy, nephropathy and atherosclerosis in both type 1 and type 2 diabetes [34,35] and also in PAH [36]. However, endothelial dysfunction is not consistently found in insulin resistance. In Zucker rats, endothelial function was impaired in the aorta and several systemic arteries [37]. In contrast, vascular reactivity and eNOS expression or phosphorylation were unchanged in hindlimb arteries [38]. Moreover, endothelial dysfunction was found in penile arteries but not in coronary arteries from obese Zucker rats in a single study [32], confirming the tissue-dependency of this effect. To our knowledge pulmonary endothelial function has not been analyzed in the context of insulin resistance. In the present experiments, the ACh-relaxation curve in conductance and resistance PA and the eNOS mRNA and protein expression were similar in obese as compared to lean rats, indicating a preserved PA-endothelial function in this model. However, our group has recently reported endothelial dysfunction in PA of type 1 diabetic rats associated to increased ROS production and increased expression of NADPH [8] as well as hyperresponsiveness to 5-HT.

In contrast to all the above described similarities between obese and lean rats, we found differences in the constrictor response in resistance but not in conductance PA from obese rats. Resistance PA showed diminished contractile responses to hypoxia, phenylephrine, KCl and 5-HT as compared to lean resistance PA, while similar responses to phenylephrine, KCl or 5-HT were found in conductance PA. In contrast, in a type 1 rat model of diabetes decreased responses were found in conductance but not in small PA [39]. Responses to vasoconstrictors have been also described to be reduced in some systemic beds from obese Zucker rats such as the mesenteric arteries [23] but enhanced in others such as the penile and coronary arteries [32]. Western blot analysis revealed no changes in the whole lung expression of 5-HT_2A_, ruling out that downregulation of 5-HT_2A _could be responsible of the reduced response to 5-HT in resistance PA.

Inducible nitric oxide synthase has emerged as a key protein in insulin resistance and obesity. Moreover, iNOS has been directly related to cardiac contractile dysfunction [40] and in vascular complications derived from insulin resistance [41,42]. We found that the contractile response to 5-HT was increased by the non selective NO synthase inhibitor L-NAME much more effectively in the obese than in the lean rats, suggesting that increased NO synthesis was responsible for the vascular hyporesponsiveness in the obese rats. Furthermore, the incubation with selective iNOS inhibitor 1400W restored 5-HT response curve suggesting that iNOS was responsible for this exaggerated NO synthesis. Since iNOS activity is primarily regulated at a transcriptional level and that once expressed the enzyme produces large amounts of NO, we investigated iNOS expression levels. The levels of iNOS mRNA tended to be higher in resistance PA from obese rats but differences did not reach statistical significance due to the high variability within our experimental samples. However the protein iNOS expression was significantly higher in obese resistance PA than in lean resistance PA. iNOS upregulation has also been found in other tissues such as the aorta, the visceral adipose tissue and the heart in the Zucker obese rats and other models of insulin resistance [40,42,43]. There are a large number of studies showing that increased expression of iNOS induced by lipopolysaccharide (LPS) is accompanied by endothelial dysfunction, as opposed to the present study. Moreover, iNOS gene deletion or pharmacological inhibition prevents LPS-induced endothelial dysfunction suggesting a cause-effect relationship [44]. However, iNOS overexpression induced by LPS is much larger (e.g. > 10 fold increase) than in the present study. More importantly, it is peroxynitrite (and probably not NO itself) produced in the reaction of iNOS-derived NO with superoxide which is responsible for endothelial dysfunction [45]. We have not measured superoxide or peroxynitrite in resistance PA, but the lack of endothelial dysfunction suggests that oxidative stress is not increased in these arteries.

## Conclusions

Herein we characterized for the first time the effects of insulin resistance in the pulmonary circulation of the obese Zucker rats. Some studies have related insulin resistance with PAH in humans and in other animal models but we did not find any of the characteristic features related with this pathology in the obese Zucker rat at the age of 17-18 weeks. However, this rat strain showed pulmonary vascular hyporesponsiveness in resistance arteries which could be prevented by inhibition of iNOS.

## List of abbreviations

ACh: acetylcholine; BMPR2: bone morphogenetic protein receptor 2; E_max_: maximum response; LV: left ventricle; PA: pulmonary arteries; PAH: pulmonary arterial hypertension; PASMC: pulmonary artery smooth muscle cells; pD_2_: negative logarithm of the molar concentration that causes 50% of the maximum response; RV: right ventricle; S: septum.

## Competing interests

The authors declare that they have no competing interests.

## Authors' contributions

JM-S performed the Western blots and electrophysiological measurements and wrote the first draft of the manuscript, CM performed the PCRs and vascular reactivity, EM measured hearts and glucose, AC and LM supervised and coordinated the study. FP-V conceived the study and wrote the final manuscript. All authors contributed to the analysis and interpretation of the data. All authors have read and approved the final submission.
